# Methodologies used by Nursing professionals in the production of educational videos: An integrative review

**DOI:** 10.1590/1518-8345.6690.3951

**Published:** 2023-06-02

**Authors:** Rafael Fernando Mendes Barbosa, Anne Ketlley Lacerda de Lima Gonzaga, Fabrine Aguilar Jardim, Karina Dal Sasso Mendes, Namie Okino Sawada

**Affiliations:** 1 Universidade de São Paulo, Escola de Enfermagem de Ribeirão Preto, Centro Colaborador de la OPS/OMS para el Desarrollo de la Investigación en Enfermería, Ribeirão Preto, SP, Brasil.; 2 Universidade Federal de Alfenas, Escola de Enfermagem, Alfenas, MG, Brasil.

**Keywords:** Nursing, Nursing Education, Instructional Films and Videos, Planning Techniques, Validation Study, Educational Technology, Enfermería, Educación en Enfermería, Películas y Videos Educativos, Técnicas de Planificación, Estudios de Validación, Tecnología Educacional, Enfermagem, Educação em Enfermagem, Filmes e Vídeos Educativos, Técnicas de Planejamento, Estudos de Validação, Tecnologia Educacional

## Abstract

**Objective:**

to evaluate the diverse scientific evidence on the methodologies used by Nursing professionals in the production of educational videos.

**Method:**

an integrative review. The search for primary studies was carried out in the CINAHL, LILACS and MEDLINE/PubMed databases. The sample consisted of 19 research studies. The methodological quality of the studies included was assessed using a tool proposed by the Johns Hopkins Nursing Evidence-Based Practice and the results were analyzed in a descriptive form.

**Results:**

the methodological stages used for the process to elaborate and make the videos include pre-production, production and post-production. The studies reveal that, for the most part, the stages were properly applied and/or described by the authors, in addition to contemplating the method adopted. However, in 14 studies there was no use of a methodological framework to ensure rigor in their conduction and in 11 presented validation by the target audience.

**Conclusion:**

the synthesis of knowledge showed that there is still a need for attention for the construction of educational videos regarding the methodological framework and validation by the target population. The rigorous execution of the methodological procedures necessary for the development of educational videos, aiming to encourage the acquisition of essential skills for the creation of high-quality teaching materials.

## Abstract (es)

**Objetivo:**

evaluar la evidencia científica sobre las metodologías que utilizan los profesionales de enfermería para producir videos educativos.

**Método:**

revisión integradora. La búsqueda de estudios primarios se realizó en las bases de datos CINAHL, LILACS y MEDLINE/PubMed. La muestra estuvo compuesta por 19 investigaciones. La calidad metodológica de los estudios incluidos se evaluó usando una herramienta propuesta por la *Johns Hopkins Nursing Evidence-Based Practice* y los resultados se analizaron de forma descriptiva.

**Resultados:**

las etapas metodológicas utilizadas para el proceso de elaboración y realización de los videos incluyen preproducción, producción y postproducción. Los estudios revelan que, en general, los autores aplicaron y/o describieron correctamente las etapas, además contemplaban el método adoptado. Sin embargo, en 14 estudios no se utilizó un marco metodológico para garantizar el rigor en su realización y en 11 presentaron validación por parte del público objetivo.

**Conclusión:**

la síntesis de conocimientos mostró que aún hay necesidad de atención en la construcción de videos educativos en cuanto al marco metodológico y la validación por la población objetivo. La ejecución rigurosa de los procedimientos metodológicos necesarios para el desarrollo de videos educativos, para fomentar la adquisición de habilidades esenciales para la creación de material didáctico de alta calidad.

## Abstract (pt)

**Objetivo:**

avaliar as evidências científicas sobre as metodologias utilizadas pelos profissionais de enfermagem na produção de vídeos educativos.

**Método:**

revisão integrativa. A busca dos estudos primários foi realizada nas bases de dados CINAHL, LILACS e MEDLINE/PubMed. A amostra foi composta por 19 pesquisas. A qualidade metodológica dos estudos incluídos foi avaliada por meio ferramenta proposta por *Johns Hopkins Nursing Evidence-Based Practice* e os resultados foram analisados de forma descritiva.

**Resultados:**

as etapas metodológicas utilizadas para o processo de elaboração e construção dos vídeos compreendem a pré-produção, produção e pós-produção. Os estudos revelaram que, majoritariamente, as etapas foram aplicadas e/ou descritas corretamente pelos autores, além de contemplar o método adotado. No entanto, em 14 estudos não houve a utilização de referencial metodológico para assegurar o rigor em sua condução e em 11 apresentaram a validação pelo público-alvo.

**Conclusão:**

a síntese de conhecimento mostrou que ainda há necessidade de atenção para a construção de vídeos educativos quanto ao referencial metodológico e validação pela população-alvo. A execução rigorosa dos procedimentos metodológicos para o desenvolvimento de vídeos educacionais, permitem fomentar a aquisição de habilidades essenciais para a criação de materiais didáticos de elevada qualidade.

**Highlights:**

**(1)** The making of videos includes the pre-production, production and post-production phases.

**(2)** Video is a powerful resource in the construction of knowledge and care practice.

**(3)** The methods for making videos guarantee the quality of the content addressed.

**(4)** Video enhances Nursing professionals’ skills in the clinical practice.

**(5)** Educational videos are essential in the training of Nursing professionals.

## Introduction

Using educational technologies to mediate health professionals’ education process regarding health interventions expands access to information, eases the teaching-learning process, promotes knowledge dissemination and causes changes in health care, through devices that favor positive perspectives in the health standards^(1-2)^.

In the Nursing education context, educational videos (EVs) have been widely used as a rich, interesting and complex tool that contributes to the promotion of education^(3-4)^. When properly developed, EVs can serve as a solid foundation to support understanding and effective reflection. However, their production requires special precautions in relation to the structuring and organization of all the information. This educational tool is used in different ways in teaching-learning environments to present motivational concepts or experiences, convey information and other applications^(5)^.

It is also noted that access to EVs, to be made available on digital platforms, contributes to reaching the target audience regardless of their geographic location and time, and enables Nursing professionals to update on the theme developed, with the possibility of positive repercussions on the assistance provided^(6)^.

Several methods have been used for the development of educational videos (EVs) and three stages are generally adopted, namely: pre-production (preparation and validation of a script and storyboard), production (video recording and editing) and post-production (evaluation of the video by the target audience)^(7-8)^.

When properly created and structured, following the methodological steps for their elaboration, EVs can become a powerful tool for building knowledge and improving the care practice. In addition to that, due to their potential as an appealing visual communication medium, EVs have aroused great interest in users^(5-6)^.

A study whose objective was to validate a video script and storyboard for an educational intervention on Nursing care aimed at syphilis prevention and management showed that the educational material produced can contribute for Nursing professionals to better understand the issues that involved the occurrence, prevention, diagnosis and treatment of syphilis^(9)^.

In addition to that, it provided users of health services with the choice of infection prevention methods, as well as with the perception of the benefits of self-care, with regard to changing behaviors and to safe sexual practices^(9)^. Other studies^(10-11)^ showed that using videos contributed to advancing knowledge in Nursing and to acquiring practical skills by Nursing students and professionals, in addition to methodologically supporting the development of other EVs in the health area. In this context, studies that develop and validate EVs on different Nursing procedures are relevant both for education and for health care. This is because they allow Nursing professionals to incorporate validated educational technologies, such as videos, in order to promote permanent and continuing education^(10,12-17)^.

Considering the methodological rigor required for the construction and elaboration of EVs, in some cases, the scientific production related to the theme has shown failures in fulfilling the methodological steps, which exerts negative impacts on the quality of the materials produced. In this sense, this review is justified by the contribution to Nursing professionals’ knowledge, through the presentation of diverse information about the methodologies used in the production of EVs. This approach aims at favoring the creation of safe and good quality educational materials, promoting the dissemination of scientific knowledge in the Nursing area.

Therefore, the objective of this study is to evaluate the diverse scientific evidence on the methodologies used by Nursing professionals in the production of educational videos.

## Method

### Type of study

The integrative review (IR) method was selected as the knowledge synthesis for this study. The stages followed were: elaboration of the research question, sampling, categorization of the studies, evaluation of the studies included in the integrative review, interpretation of the results and synthesis of the results^(18)^.

This integrative review was registered on the Open Science Framework (OSF) platform on May 20^th^, 2022, and the protocol can be accessed at https://osf.io/rh5wa, with the following DOI identifier: 10.17605/OSF.IO/RH5WA ^(19)^.

### Locus

The integrative review was conducted in the municipality of Passos, located in the state of Minas Gerais, Brazil.

### Period

The study period was from June 2022 to January 2023.

### Population

The guiding question of this study was defined as follows: “Which is the diverse evidence available in the literature about the methodologies used by Nursing professionals in the production of educational videos?” In order to formulate the question, the PCC acronym (Population, Concept and Context) was adopted, where P = Nursing professionals; C = Methodologies used; and C = Production and validation of educational films and videos.

### Selection criteria

The materials considered eligible were primary studies published between 2017 and 2022 in English, Portuguese or Spanish, which addressed the use of methodologies for the production of educational videos involving Nursing professionals. Case studies, editorials, theses and dissertations were excluded, as well as those materials that did not clearly present the methodological process for developing educational videos. Updated references were used to increase credibility of the results.

### Sample definition

Three relevant databases in the Health and Nursing areas were selected to search for primary studies, namely: PubMed, Cumulative Index to Nursing and Allied Health Literature (CINAHL) and *Literatura Latino-Americana e do Caribe em Ciências da Saúde* (LILACS).

The three items from the PCC acronym were used to create different combinations of controlled descriptors, keywords and AND and OR Boolean operators, in order to obtain the search strategies in the databases. In the PubMed database, the controlled descriptors were selected from the Medical Subject Headings (MeSH), and the search strategies adopted were as follows: ((“Nursing”[Mesh] OR “Nursing” OR “Nursings”)) AND ((“Instructional Film and Video” [Publication Type] OR “Instructional Film and Video” OR “Instructional Films and Videos” OR “Instructional Films and Video” OR “Instruction” OR “Audio-Video Demonstration” OR “Audiovisual Demonstration” OR “Video-Audio Demonstration”)) AND ((“methods” [Subheading] OR “methods” OR “techniques” OR “procedures” OR “methodology” OR “Planning Techniques”[Mesh] OR “Planning Techniques” OR “Planning Technique” OR “Planning Technic” OR “Planning Technics” OR “Planning Methodologies” OR “Planning Methodology” OR “Planning Theories” OR “Planning Theory” OR “Validation Study” [Publication Type] OR “Validation Study” OR “Validation Studies”)).

The search strategies in the CINAHL and LILACS databases were similar, but they used the vocabularies specific to each one: CINAHL Headings and Descriptors in Health Sciences (*Descritores em Ciências da Saúde*, DeCS), respectively. The search strategies were implemented in the databases selected on June 3^rd^, 2022.

After applying the search strategy in each of the databases selected, the results obtained were exported to the EndNote X7 reference manager software (Desktop version)^(20)^, where duplicate studies were removed. The articles were screened in the Rayyan^(21)^ web app, with the objective of selecting studies that met the previously established inclusion criteria.

During the first selection phase, the titles and abstracts of the studies were read to assess whether they met the eligibility criteria of the integrative review. In a second phase, the articles selected were read in full by two reviewers who worked independently and blindly, based on the review question and the eligibility criteria established. If there was any disagreement between the reviewers, a third one with knowledge in the area was consulted to resolve it.

In addition to the search in the databases, a manual search was conducted by the reviewer in the bibliographic references of the primary studies selected in the integrative review. The search and selection process for primary studies took place between June and August 2022.

### Data collection

The data from the studies were collected through an adapted script^(22)^, which included the following information: reference and year of publication, objective, methodological characteristics (study design according to the nomenclature used by the authors and sample) and main results (methodologies used for the production and validation of educational videos). This data collection stage took place between September and November 2022, with the participation of two independent reviewers, who discussed any disagreements in meetings to reach consensus.

Allocation of the studies by their knowledge area was based on the classification table of the Council for Scientific and Technological Development (CNPq), which adopts a given knowledge tree as support for the development of its papers. Nursing is reflected in its current tree as follows: Adults’ and Older Adults’ Health Nursing; Women’s Health Nursing; Children’s and Adolescents’ Health Nursing; Mental Health Nursing; Collective Health Nursing; Fundamental Nursing; and Nursing in Administration and Management^(23)^.

### Data treatment and analysis

The data were analyzed qualitatively, with a synthesis of the diverse evidence from the primary studies performed in a descriptive manner. The type of study was classified based on the designation established by the authors of the studies included in the review.

For the critical evaluation stage, it was decided to assess the methodological quality of the primary studies included in the sample, using the tool proposed by the Johns Hopkins Nursing Evidence-Based Practice^(24)^ by two reviewers, also independently. Such evaluation was carried out considering the appropriate tool for the type of design included (quantitative, qualitative or mixed), which presents the “Yes”, “No” or “Not Applicable” answers. Before starting the critical evaluation of the studies, decisions about judgment were agreed upon between the reviewers and a third reviewer was consulted in case of conflicts in the evaluation between the first two reviewers.

The studies included were categorized according to their methodological quality, considering the following as high quality: consistent and generalizable results; sufficient sample size for the study design; adequate control; definitive conclusions; consistent recommendations based on a comprehensive literature review that includes full reference to the scientific evidence; as good quality: reasonably consistent results; sufficient sample size for the study design; some control; fair definitive conclusions; reasonably consistent recommendations based on a fairly comprehensive literature review that includes some reference to the scientific evidence; and as low quality: limited evidence with inconsistent results; insufficient sample size for the study design; impossibility to draw conclusions^(24)^.

Considering that it is fundamental to unite methodological quality and strength of the evidence for decision-making in the clinical practice, the studies evaluated were classified according to their level of evidence, as per the evidence hierarchy guide of the Johns Hopkins Nursing Evidence-Based Practice^(24)^, as follows: Level 1 – 1.a) Experimental study, Randomized Controlled Trial (RCT); 1.b) Explanatory mixed-methods project that includes only one Level 1 quantitative study; 1.c) Systematic review of Randomized Clinical Trials (RCTs), with or without meta-analysis; Level 2 – 2.a) Quasi-experimental study; 2.b) Explanatory mixed-methods project that includes only one Level 2 quantitative study; 2.c) Systematic review of a combination of RCTs and quasi-experimental studies, or quasi-experimental studies only, with or without meta-analysis; Level 3 – 3.a) Systematic review of a combination of RCTs, quasi-experimental and non-experimental studies, or non-experimental studies only, with or without meta-analysis; 3.b) Exploratory, convergent or multiphase mixed-methods studies; 3.c) Explanatory mixed-methods project that includes only one Level 3 quantitative study; 3.d) Qualitative study; 3.e) Systematic review of qualitative studies with or without meta-synthesis. Evidence of non-research: Level 4 – Opinion from respected authorities and/or nationally recognized experts’ committees or consensus panels based on scientific evidence, which includes: clinical practice guidelines and consensus panels/position statements; and Level 5 – Based on experimental evidence and not related to research, which includes: scoping reviews; integrative reviews; literature reviews; quality improvement, program or financial evaluation; case reports; nationally recognized experts’ opinion based on experimental evidence^(24)^.

## Results

**Figure 1 -:**
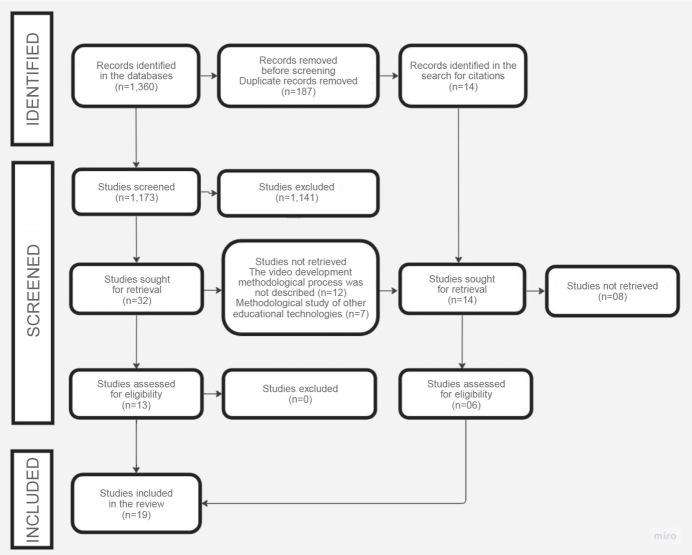
Selection flowchart corresponding to the studies included in this integrative review. Passos, MG, Brazil, 2022

Figure1 shows the flowchart corresponding to the selection process of primary studies included in this integrative review. The initial search in the databases identified 1,360 records, of which 32 were selected for full reading, following the eligibility criteria. After applying the inclusion and exclusion criteria, 19 primary studies were considered eligible and included in the final sample.

Figure2 presents the descriptive synthesis of the primary studies selected, including information on author, year of publication, objective, type of study and sample.

**Figure 2 -:** Synthesis table of the studies included to comprise the final sample of this integrative review (author, year of publication, type of study, sample and objective). Passos, MG, Brazil, 2022

Primary study/ Year of publication	Type of study/Sample	Objective
Lopes, et al.^(10)^ 2020	Methodological study (authors) Sample: n=7	To develop and validate a video on the bed bath technique aimed at Nursing professionals and students.
Faleiros, et al.^(11)^ 2019	Methodological study (authors) Sample: n=18	To develop and validate an educational video using 3D technology and avatars, which aims at improving learning of the clean intermittent self-catheterization technique in female and male patients.
Yang, et al.^(12)^ 2022	Methodological and descriptive study (authors) Sample: n=5	To develop and evaluate content validity and usability of a 360-degree panoramic video to aid in physical examination teaching and practice.
Antoniolli, et al.^(13)^ 2021	Methodological study (authors) Sample: n=7	To develop and validate digital educational resources to promote health and safety at work for professionals working in Primary Health Care.
Silva, et al.^(14)^ 2017	Methodological study (authors) Sample: n=5	To develop and validate an educational video addressing the foot reflexology technique.
Caetano, et al.^(15)^ 2021	Methodological study (authors) Sample: n=8	To produce and validate an educational video that shows the oscillometric technique for blood pressure indirect measurement, aiming to support the actions of blood pressure screening programs in Brazil.
Sanguino, et al.^(16)^ 2021	Methodological study (authors) Sample: n=28	To create and validate an educational video that addresses management of cardiopulmonary arrest in children due to respiratory failure.
Ferreira, et al.^(17)^ 2015	Methodological and descriptive study (authors) Sample: n=25	To develop and validate an educational video in digital format on the dressing technique for uncuffed, non-tunneled and short-term central venous catheters, aimed at hospitalized adult patients.
Razera, et al.^(25)^ 2019	Methodological and descriptive study (authors) Sample: n=6	To elaborate and describe the construction process of an educational video intended to instruct on the necessary care measures during the postoperative period of primary cheiloplasty and palatoplasty surgeries.
Nazario, et al.^(26)^ 2021	Methodological study (authors) Sample: n=19	To develop and evaluate the effectiveness of an educational video aimed at encouraging the active participation of the family in acute pain relief in infants.
Rosa, et al.^(27)^ 2019	Methodological study (authors) Sample: n=7	To elaborate and validate an educational resource in video format for individuals and families dealing with the experience of colostomy and cancer.
Rodrigues Junior, et al.^(28)^ 2017	Methodological study (authors) Sample: n=14	To elaborate an educational video with the objective of early detecting visual difficulties in students.
Campos, et al.^(29)^ 2021	Methodological study (authors) Sample: n=20	To develop and validate an educational video, with animation resources, targeted at family caregivers to provide guidance on the home bath procedure for full-term newborns.
Grave, et al.^(30)^ 2021	Methodological and descriptive study (authors) Sample: n=35	To develop and validate educational videos on the health needs associated with chemotherapy treatments.
Galindo-Neto, et al.^(31)^ 2019	Methodological study (authors) Sample: n=22	To develop and evaluate an educational video for teaching cardiopulmonary resuscitation techniques to deaf students.
Braga, et al.^(32)^ 2014	Methodological and descriptive study (authors) Sample: n=5	To describe the development and validation process of an educational video designed to guide patients undergoing chemotherapy treatments on proper oral hygiene practices.
Lima, et al.^(33)^ 2017	Methodological study (authors) Sample: n=12	To elaborate and validate an educational video to provide guidance about proper use of clean intermittent catheterization to parents of sick children.
Rossi, et al.^(34)^ 2019	Methodological study (authors) Sample: n=79	To develop and validate educational videos to address female and male indwelling catheterization techniques in adult patients.
Campoy, et al.^(35)^ 2018	Methodological study (authors) Sample: n=19	To develop and validate an educational video to train individuals with neurogenic bowel in bowel emptying maneuvers, as part of the bowel rehabilitation process.

Figure3 contains diverse information about the main results and conclusions of the studies, knowledge area, methodological quality and level of evidence.

**Figure 3 -:** Synthesis chart of the studies included to comprise the final sample of this integrative review (main results and conclusions, knowledge area, methodological quality and level of evidence). Passos, MG, Brazil, 2022

Primary study	Main Results/Conclusions	Knowledge area	Johns Hopkins	LE
Lopes, et al.^(10)^	Six script validation rounds were carried out by the experts. Subsequently, the students evaluated the video and all scores were equal to or greater than four, with a high percentage of maximum scores, varying from 77% to 95%. It was observed that the video was effective in contributing to professional training and in improving Nursing students’ knowledge and skills.	Fundamental Nursing	High quality	IIIc
Faleiros, et al.^(11)^	The agreement level between the evaluators in relation to the questions assessed in the validation process of the educational video was 97.4%. The video was created with the objective of teaching intermittent bladder self-catheterization to people with neurogenic bladder, filling a gap in the availability of audiovisual educational resources for this purpose in the Brazilian context.	Fundamental Nursing	High quality	IIIc
Yang, et al.^(12)^	The mean Content Validity Index (CVI^*^) referring to relevance of the content was 0.97, whereas it was 0.98 and 0.95 for convenience, respectively, according to the validation carried out by Nursing professors. Nursing students considered the educational video as a positive learning method for the physical examination. In addition to that, it provided a physical assessment technique that was easy to employ and useful for the practice in real situations.	Fundamental Nursing	Good quality	IIIc
Antoniolli, et al.^(13)^	The contents were structured in storyboards and organized into seven digital educational resources. The digital resources were validated by professors, occupational health and safety professionals and Primary Health Care professionals, reaching CVI^*^ values from 0.88 to 0.96. Using videos as a learning tool proves to be effective in promoting strategic actions to promote health and safety at work among Primary Health Care professionals.	Nursing in Administration and Management	High quality	IIIc
Silva, et al.^(14)^	Validation of the video for the target population, carried out by experts, proved to be adequate and appropriate. The students positively evaluated understanding and scope of the video content, as well as its clarity and objectivity. The video meets the necessary criteria for its application in the population of interest, standing out for its clarity and objectivity.	Adults’ and Older Adults’ Health Nursing	Good quality	IIIc
Caetano, et al.^(15)^	Validation of the video for training health professionals interested in blood pressure screening programs in different Brazilian communities obtained adequate agreement from the experts (CVI^*^=0.94). The video was considered as an appropriate strategy to teach the oscillometric technique for indirect blood pressure measurement among health professionals.	Public Health Nursing	High quality	IIIc
Sanguino, et al.^(16)^	After being validated by Nursing specialists and students, the educational video developed to teach management of pediatric cardiopulmonary arrest due to respiratory failure reached more than 80% agreement in its items. This innovative digital resource is considered suitable for use by Nursing students, constituting a valuable learning tool.	Children’s and Adolescents’ Health Nursing	High quality	IIIc
Ferreira, et al.^(17)^	The script approval percentage among the nurses was 97.2%, with 96.1% for the video. The sum of the answers related to relevance, environment and verbal language resulted in 100%. The video was validated and considered adequate to be used as a pedagogical resource in Nursing courses and related areas, providing students with a simulated clinical experience that can contribute to their professional training.	Fundamental Nursing / Adults’ and Older Adults’ Health Nursing	High quality	IIIc
Razera, et al.^(25)^	The agreement index among the judges specialized in communication and health was 98%, validating the video as an effective communication tool. The language used in the video was considered easy to understand and suitable for the target audience, and the audiovisual resources were properly employed. The video has the potential for wide broadcast and distribution, as well as for encouraging positive attitudes from caregivers towards post-operative care after cheiloplasty and palatoplasty surgeries.	Medical-Surgical Nursing	High quality	IIIc
Nazario, et al.^(26)^	The results of the video validation by expert judges showed 90% agreement. Family members and pregnant women were invited to evaluate the video as an educational technology for learning and both groups positively assessed the health education strategy. The video proved to be an effective tool to assist in acute pain relief in newborns and can be considered an option for educational actions in health.	Children’s and Adolescents’ Health Nursing	High quality	IIIc
Rosa, et al.^(27)^	After the evaluation by expert judges, the script content obtained a CVI^*^ of 0.99. Subsequently, the video was validated by the target audience and by the same expert judges, with a CVI* of 1. These results confirm quality of the content and adequacy of the video for educational use in Nursing, especially in the care of people with a stoma and their families, highlighting its potential as an empowering resource.	Adults’ and Older Adults’ Health Nursing	High quality	IIIc
Rodrigues Junior, et al.^(28)^	In general, the video script was approved by the experts, obtaining a CVI^*^ value ≥ 0.8, although 57.1% of them suggested some modification. The educational video is considered a valuable learning resource for professors, parents and family members in identifying behaviors that indicate vision difficulties in students.	Children’s and Adolescents’ Health Nursing	High quality	IIIc
Campos, et al.^(29)^	The educational video on the home bath for full-term newborns was submitted to the evaluation by expert judges in the Nursing and Social Communication areas. The overall mean CVI* value was 0.99 (99%), indicating a satisfactory assessment in relation to all items, which obtained values above 0.8 (80%). Therefore, the educational video was validated satisfactorily.	Children’s and Adolescents’ Health Nursing	High quality	IIIc
Grave, et al.^(30)^	The results of the validation by experts indicate that the total CVI^*^ and the items from the evaluation criteria were greater than 0.80 and presented a 95% confidence interval, whose lower limit was also above 0.80. Therefore, the videos proved to be effective in stimulating favorable behaviors for maintaining health, providing diverse information for symptom self-management and reduction, which can lead to a significant improvement in the patients’ quality of life.	Adults’ and Older Adults’ Health Nursing	High quality	IIIc
Galindo-Neto, et al.^(31)^	During content validation of the video storyboard, the experts reached a minimum agreement of 86% in relation to the evaluated items. The video represents a viable technological solution for nurses and other health professionals who wish to offer content related to cardiopulmonary resuscitation to deaf students.	Adults’ and Older Adults’ Health Nursing	High quality	IIIc
Braga, et al.^(32)^	The results of the video validation by the experts reached scores equal to or greater than 80%. In the evaluation carried out by the patients regarding understanding of the video, a score of 9.83 was obtained. The educational video is considered as a useful resource for health professionals who wish to provide diverse information and guidelines on proper oral hygiene for patients undergoing cancer treatment.	Adults’ and Older Adults’ Health Nursing	High quality	IIIc
Lima, et al.^(33)^	The technical judges presented results of 0.745, 0.771 and 0.777, with p<0.0001, respectively, for the assessments of language clarity, relevance for the practice and theoretical relevance of the video. The intraclass correlation coefficient for all the categories assessed was 0.768, considered reasonable by the experts. Thus, it was concluded that the video is a relevant resource for communication and education of parents and caregivers, assisting in the care of children subjected to clean intermittent catheterization.	Children’s and Adolescents’ Health Nursing	High quality	IIIc
Rossi, et al.^(34)^	The video script validation by the specialists obtained satisfactory results. In addition to that, the final version was submitted to an evaluation by 71 students attending first year of the undergraduate Nursing course, obtaining significant agreement (p<0.0001). The creation of videos that address Nursing techniques, such as catheterization, can encourage the development of videos for other procedures and contribute to students’ learning, as well as help professors in their educational practices.	Fundamental Nursing	High quality	IIIc
Campoy, et al.^(35)^	The script and storyboard validation was carried out by specialists in the theme and technicians, obtaining approval rates of 94% and 100%, respectively. Subsequently, the video was rated positively by individuals with neurogenic bowel, achieving a 100% approval rate. The video was considered valid and can significantly contribute to the improvement of Nursing care, focusing on the rehabilitation of individuals with neurogenic bowel and their caregivers.	Fundamental Nursing / Adults’ and Older Adults’ Health Nursing	High quality	IIIc

^*^CVI = Content Validity Index

The methodological stages used for the elaboration and construction process of the videos include pre-production, production and post-production. Figure4 presents the implementation of the methodological stages adopted in the studies included in this review, represented by the methodologies used for the production and validation of EVs and the methodological framework used, revealing that most of the methodological stages were properly applied and/or described by the authors, in addition to considering the method adopted. However, in 14 studies there was no use of methodological framework to ensure rigor in its conduction. Regarding the evaluation, 11 studies were validated by target audience.

**Figure 4 -:** Implementation of the methodological stages covered in the studies included in this integrative review. Passos, MG, Brazil, 2022

Primary study	Stages					Methodological framework
Lopes, et al.^(10)^	Pre-production: development of a script and storyboard.	Production: based on the validated script and storyboard.	Post-production: video editing and inclusion of the audio.	Evaluation by the target audience.	-	Fleming, Reynolds, Wallace, 2009^(8)^.
Faleiros, et al.^(11)^	Pre-production: elaboration and validation of the script and storyboard.	Video production.	Post-production: validation of the video by expert judges.	-	-	Does not apply.
Yang, et al.^(12)^	Review of the literature on the theme.	Elaboration of the script, rehearsal of actors, filming, editing and post-production.	Evaluation by the target audience.	-	-	Does not apply.
Antoniolli, et al.^(13)^	Creation and elaboration of the storyboards.	Validation of the videos by specialists.	-	-	-	Does not apply.
Silva, et al.^(14)^	Pre-production: creation and elaboration of the script and storyboard and validation by experts.	Production based on the validated script and storyboard.	Post-production: editing and validation of the video by experts.	Assessment of the understanding and scope of the content by the target audience.	-	Fleming, Reynolds, Wallace, 2009^(8)^.
Caetano, et al.^(15)^	Pre-production: development of a script and storyboard.	Content validation by experts.	Final production, video recording and editing.	-	-	Does not apply.
Sanguino, et al.^(16)^	Elaboration and validation of a clinical case.	Production based on the creation of a script and storyboard based on a fictitious clinical case.	Validation/Evaluation of the educational video by the target audience.	-	-	Fleming, Reynolds, Wallace, 2009^(8)^.
Ferreira, et al.^(17)^	Elaboration and validation of the script and creation of the storyboard.	Production of the video.	Video validation by experts and technical judges.	-	-	Does not apply.
Razera, et al.^(25)^	Analysis and planning: definition of topics, objectives, content and target audience.	Modeling: content preparation and organization.	Implementation: process to create and produce the project.	Evaluation and maintenance: tests, corrections and validation by the target audience.	Distribution: dissemination or disclosing phase of the material.	Falkembach, 2005^(7)^.
Nazario, et al.^(26)^	Elaboration of the video with creation of the script and storyboard.	Validation of the video by expert and communication/audiovisual judges.	Evaluation by the target audience.	-	-	Does not apply.
Rosa, et al.^(27)^	Construction of the video content script.	Validation of the script, by expert judges.	Development of the educational video.	Evaluation by the target audience.	-	Does not apply.
Rodrigues Junior, et al.^(28)^	Pre-production: elaboration and validation of the script.	Video production.	Post-production: video editing.	-	-	Does not apply.
Campos, et al.^(29)^	Literature review on the theme.	Elaboration of a script.	Creation of the video based on the prepared script.	Video validation by expert judges.	Adaptations to the video requested in the validation.	Does not apply.
Grave, et al.^(30)^	Identification of the patient’s health needs related to the chemotherapy treatment.	Creation of the videos.	Validation by expert judges.	Adequacy of the educational videos.	-	Does not apply.
Galindo-Neto, et al.^(31)^	Pre-production: planning and storyboard design.	Production: creation of the video.	Post-production: validation of the video by the target audience.	-	-	Does not apply.
Braga, et al.^(32)^	Pre-production: script preparation.	Production: script validation and development of the video.	Post-production: editing and validation by experts to evaluate the video.	Verification of the extent to which the target audience understands the video.	-	Does not apply.
Lima, et al.^(33)^	Pre-production: script development, storyboard creation and validation by the judges.	Production: filming.	Post-production: editing and finalizing the video.	-	-	Fleming, Reynolds, Wallace, 2009^(8)^.
Rossi, et al.^(34)^	Pre-production: script development and validation.	Production: video recording from the validated script and storyboard.	Evaluation by the target audience.	-	-	Does not apply.
Campoy, et al.^(35)^	Writing of the script and storyboard.	Validation of the script and storyboard.	Production of the educational video from the validated script and storyboard.	Pilot study: evaluation by the target audience.	-	Does not apply.

## Discussion

The elaboration of EVs aims at disseminating and standardizing care practices, through the production of essential contents for knowledge management and improvement. This is an effective, low-cost, simple and appealing technique, recommended for the continuous and permanent education of professionals, patients and caregivers^(10,36-37)^, in order to develop critical thinking and promote health, in addition to generating immediate behavioral changes.^(17)^. EVs are configured as a teaching instrument that brings the educational environment closer to everyday relationships, using language​and codes that are understandable to the population^(38)^.

The studies^(10-17,25-35)^ identified in this review synthesized the diverse evidence related to the methodologies used by Nursing professionals in the production of EVs and found that the methodological steps most reported in the primary studies involved the pre-production stages (creation and elaboration of the script and storyboard), production (validation and production of the video from the validated script and storyboard) and post-production (evaluation by the target audience).

In the EV production and validation process, the methodology adopted for their development is essential to guarantee the quality of the content addressed. In addition to that, it is important to validate the instrument with experts who have specialized knowledge on the subject matter, in order to help reduce the possibility of inaccurate results or biased measurements that might lead to incorrect conclusions. This will result in more sophisticated instruments that are effectively used by the target audience^(39)^. Above all, the number of judges and their selection criteria exert a positive impact on the development of methodological studies and must be chosen rigorously^(40)^. The literature recommends from six to 20 judges for content validation^(41)^, which corroborates the number of judges selected among the studies included^(10-11,13,15-17,25-31,33-35)^ and is in line with other studies surveyed^(9,29,42)^ except for^(12,14,32)^, which revealed fewer judges than recommended, showing weaknesses in the stage to select the evaluators.

Due to their ability to adapt to different contexts and be applied in different Nursing areas, EVs have been widely used as an effective strategy for teaching and acquiring skills by professionals in the field. This review highlights studies conducted exclusively by Nursing professionals^(10-17,25-35)^ and produced in Brazil^(10-11,13-17,25-35)^. It is known that health education and the production of studies involving Nursing professionals in Brazil receive special attention, given the need to meet the teaching demands and changes in the students’ profiles, with the incorporation of new educational tools^(43)^, EVs among them.

In a collaborative and constructivist approach, using EVs as an educational tool can promote changes in the academic context, directing the training of health professionals to the current reality of the health-disease process in the country and encouraging the development of critical skills and commitment with the health of the population^(4,44)^. In addition to that, when compared to written language, EVs exert a greater impact on learning and allow for a wider geographic distribution^(4)^.

For the development of EVs, it is necessary to build material with a pedagogical purpose based on diverse scientific evidence so that it is validated, ensuring that the objectives for which it is built are met^(39)^. Choice of the contents for the studies was based on national and international guidelines, as well as on relevant evidence on the topic^(10-17,25-35)^, in addition to statements from experts’ associations^(11,31)^, book publications^(12)^ and informative materials^(27)^.

It was observed that the methodological framework was only adopted in some studies^(10,14,16,25,33)^. It is emphasized that using a framework for the development of educational videos, through the various stages covered, is fundamental to orient the creative process, as its use guides the content to be developed. Furthermore, this process establishes the connection between theory and practice, establishing a set of norms, procedures, techniques and analysis tools that define the ideal pattern for creating system projects or educational apps^(7)^.

The synthesis herein presented evidences that the agreement level between the judges was reached, calculated using the Content Validity Index (CVI) for each item of the instruments, which corresponds to the proportion (in %) of judges who expressed the “I agree” or “I partially agree” opinions in relation to the total number of judges for validation of the content of the scripts and storyboards and subsequent elaboration of the videos in each study^(10-17,25-35)^, proving to be adequate and appropriate for application in the population of interest.

Ensuring the understanding and appeal of the EVs produced to train individuals with special needs requires obtaining agreement between specialists and the target population regarding the evaluation items used, such as concept, clarity, content, environment, order of speeches and language. This measure contributes to validation of the content produced and to its effective application in the target audience. The results of previous studies have shown high agreement levels among specialists, meeting the minimum standards required in each study, which reinforces validity of the content produced for educational purposes^(10-17,25-35)^.

As for the weaknesses found in the studies analyzed, non-evaluation of the educational video by its target audience stands out^(11,13,15,17,28-30,33)^, as well as non-disclosure of the selection criteria for the evaluators^(17,32)^. Both weaknesses can compromise the transparency process in the production and validation of EVs.

In view of the diversity of methodologies used for the production and validation of EVs in the articles listed in this review, it becomes necessary to conduct more studies regarding the organization of this process, that is, standardization of these methodologies in order to guarantee quality and safety in the process of production and validation of educational material. The videos of the studies included in this review presented different themes, most of them centered on health education for the target audience, mainly patients, family members and/or caregivers^(11,25,27-33,35)^, whereas the videos produced in other studies addressed topics related to the teaching of procedures for Nursing students and/or professionals^(10,12-17)^.

In this context, it is remarkable that the technologies can contribute to the Nursing field, used both for educational and care purposes. The use of educational technologies, especially EVs, has been recognized as a didactic tool that combines several elements such as images, text and sound, in a single knowledge promotion object^(45-46)^. Thus, EV use can improve Nursing professionals’ skills in the clinical practice, in addition to teaching fundamental skills for care, as it assists in learning and promotes Nursing students’ performance and satisfaction^(47)^.

Most of the studies presented high quality and, for the most part, Level of evidence IIIc, although few used a methodological framework to guide the stages required for the production of EVs. It is remarkable that the use of this technology has become an increasingly present and necessary practice in the educational and care spheres, whether in the training of Nursing professionals or in health education for the population and, corroborating this trend, Brazilians nurses have assumed a prominent position in the production of this type of content.

The creation of EVs by Nursing professionals is an essential practice to contribute to health education for the general population, in addition to strengthening professional training and continuing education in the area. As health professionals, nurses play a fundamental role in health promotion, which reinforces the importance of new studies targeted at using videos as an educational strategy to improve health care quality.

This integrative review contributes to advancing scientific knowledge for the health area, especially for Nursing, as the results presented favor the production of knowledge regarding EV use and provide subsidies for the acquisition of skills in conducting other studies. It also emphasizes the importance of establishing standardized methodologies that aim at guaranteeing the quality of the materials produced, which can contribute benefits to the clinical practice, from their use in Nursing education to guidelines for patients, family members and/or caregivers.

As limitations of this study, it is worth noting the restriction on the number of databases selected to search for studies, as well as the publication period comprising the last five years and the sole inclusion of studies published in three languages: Portuguese, English and Spanish.

## Conclusion

Considering the diverse evidence synthesized on the methodologies used by Nursing professionals in the production of educational videos, it is concluded that the main stages taken for the production of videos in most of the studies involved pre-production (preparation and validation of a script and storyboard), production (video recording and editing) and post-production (evaluation of the video by the target audience).

The synthesis of knowledge showed that there is still a need for attention for the construction of educational videos regarding the methodological framework and validation by the target population. The rigorous execution of the methodological procedures necessary for the development of educational videos, aiming to encourage the acquisition of essential skills for the creation of high-quality teaching materials.
